# Characterization of Phenolic Compounds and Antiproliferative Effects of *Salvia pomifera* and *Salvia fruticosa* Extracts

**DOI:** 10.3390/molecules24162921

**Published:** 2019-08-12

**Authors:** Antonios Koutsoulas, Martina Čarnecká, Jiří Slanina, Jaroslav Tóth, Iva Slaninová

**Affiliations:** 1 Department of Pharmacognosy and Botany, Faculty of Pharmacy, Comenius University in Bratislava, Odbojárov 10, 83232 Bratislava 3, Slovak Republic; 2 Department of Biochemistry, Faculty of Medicine, Masaryk University, Kamenice 5, Building A16, 62500 Brno, Czech Republic; 3 Department of Biology, Faculty of Medicine, Masaryk University, Kamenice 5, Building A6, 62500 Brno, Czech Republic

**Keywords:** 12-*O*-methylcarnosic acid, cancer, carnosic acid, cytotoxicity, cell cycle, melanoma, microtubules, LC-MS, *Salvia fruticosa*, *Salvia pomifera*

## Abstract

The phenolic compounds of methanolic extracts of *Salvia pomifera* and *Salvia fruticosa* were identified by liquid chromatography tandem mass spectrometry. Carnosic acid and its metabolite carnosol were the most abundant terpene phenolic compounds of *S. fruticosa*, while they were completely absent in *S. pomifera*. The main terpene phenolic constituent of *S. pomifera* was 12-*O*-methylcarnosic acid and its mass/mass fragmentation pathway was explained. The detailed mechanism of carnosic acid oxidation to carnosol was suggested. The effects of *Salvia* extracts and/or carnosic acid, the main diterpene phenolic component of *S. fruticosa*, on the proliferation and cell cycle of two melanoma cell lines (A375, Mel JuSo) and human fibroblast cell line (HFF) were investigated by MTT assay, PI-exclusion assay and flow cytometry cell cycle analysis. Extract of *S. fruticosa* more efficiently than *S. pomifera* extract reduced the proliferation of the human melanoma cells. Carnosic acid showed the most significant effect. The first evidence that carnosic acid affects microtubule dynamics and arrests the cell cycle in the G2/M phase was provided. Collectively, our results demonstrate that these two *Salvia* species are plants of medicinal interest with perspective for further investigation. Carnosic acid could be the compound responsible for the biological activities of *S. fruticosa* extracts.

## 1. Introduction

**Figure 1:**
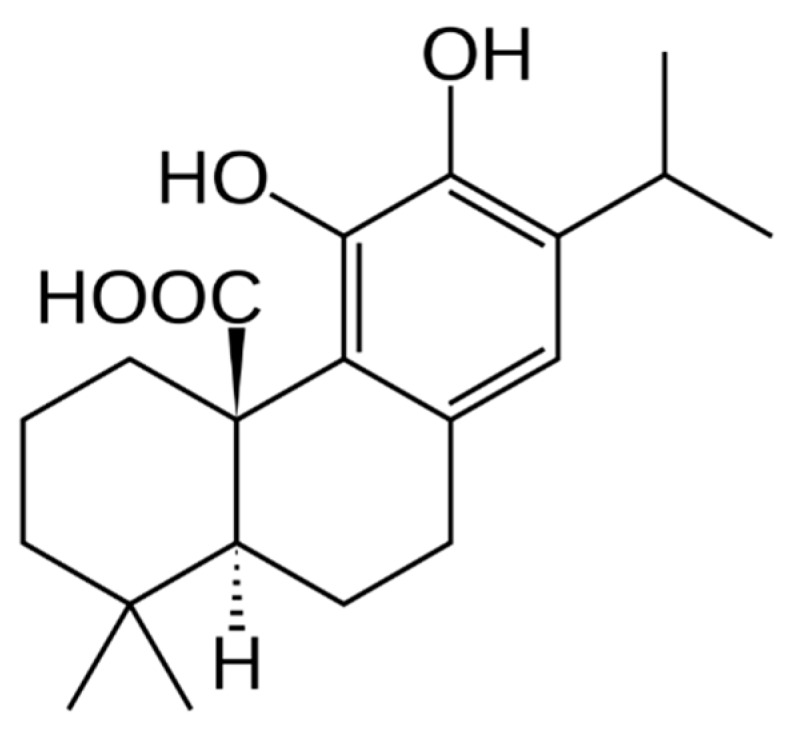
Structure of carnosic acid.

The genus *Salvia* L. (Lamiaceae) comprises a large group of plants with more than 900 different plant species. *Salvia* plants are predominantly found in the East and Central Mediterranean region. Of those, only a few taxa have medicinal and commercial value [1,2,3,4]. Nevertheless, many of these species are used in traditional medicine and several previous studies identified various biologically active compounds and explored the pharmacological properties. These studies revealed anti-diabetic, anti-oxidant, anti-inflammatory as well as anti-tumor activities of plant extracts and isolates. These effects were mainly attributed to plant phenolics, flavonoids, coumarins, terpenes and carotenoids, such as rosmarinic acid (phenolic) and carnosic acid (CA, terpene) [3,4,5,6,7]. In this study, we focused on two *Salvia* species, *Salvia fruticosa* Mill. (SF) and *Salvia pomifera* L. (SP), which are used in Crete as traditional medicines, however clinical evidence for these traditional indications in accordance with EBM (Evidence Based Medicine) is lacking. In particular, SF plants, which are the most important sage in the United States, have shown a cytotoxic effect in several cancer cell lines in vitro, including human colon, pancreatic, breast, and cervical carcinoma and also leukemic and melanoma cell lines [2,3,4,6,7,8,9]. As with other *Salvia* species, SF chemoprotective properties are cell line dependent and several mechanisms for the molecular pathways’ activation have been proposed, such as DNA damage prevention, enhanced DNA repair, cell proliferation inhibition and apoptosis induction [5,10]. For these reasons, the mechanism of action of these plants is not clear yet and several studies have to be performed in order to shed light upon its potent anticancer role. Despite the fact that SP exhibits a high antioxidative capacity, it has not attracted scientific interest so far, and there are no in vitro or in vivo data available on its role in cancer therapy [11]. CA (Figure 1) is a benzenediol diterpene with a characteristic phenolic group. It is often found in several *Salvia* plants and has a natural anti-oxidative role. It seems that CA is not distributed evenly in plant tissues but rather is found predominantly in the aerial parts. It has also been shown that the amount of CA in plants varies based on the ontogenetic stage of the plant and the environmental conditions [12,13,14,15]. Several studies have shown that CA exhibits interesting biological effects such as anti-inflammatory and anti-oxidant activities. It is of particular interest that it showed anti-tumor activity in several cell lines including leukemic and melanoma cells as well as in breast, prostate, lung, and liver cancer cell lines. The mechanism of action through which CA affects cancer cells is not clear but it seems that it is cell line dependent [13,16,17,18,19]. For example, it seems that CA leads to autophagy activation in human hepatoma cells but the same molecule activates apoptosis and cell cycle arrest in prostate cancer cells [13,20]. Especially in melanoma cells, previous studies have shown that CA seems to block cell migration of B16F10, an aggressive melanoma cell line by inhibiting epithelial-mesenchymal transition (EMT). This inhibition subsequently resulted in anoikis activation, which is a form of apoptosis [18]. In another study, CA activated the KEAP1/NRF2 axis inducing NQO1 expression. In this way, CA could enhance the toxicity of other anti-cancer drugs in melanoma treatment [12]. Melanoma is an aggressive skin cancer type that develops from melanocytes, which are responsible for melanin pigmentation. Though primary melanoma can be successfully surgically treated in most cases, therapy options of metastatic melanoma are limited [21]. In the present study, we focused on the ability of two *Salvia* extracts to affect melanoma cells in vitro and compared their effect with CA, the primary diterpene phenolic component of SF but not SP.

## 2. Results

### 2.1. Identification of Phenolic Compounds in SF and SP by HPLC–ESI-QTOF-MS

**Figure 2:**
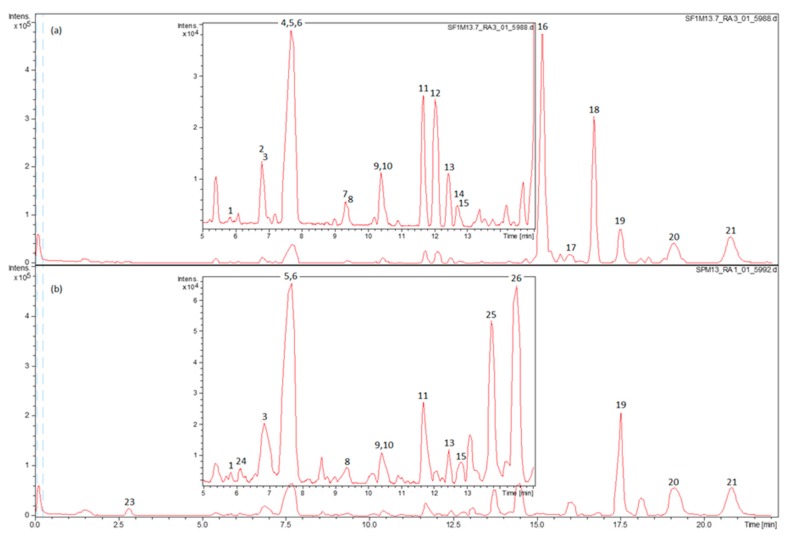
The base peak chromatograms (BPC) of methanolic extract of (**a**) *Salvia fruticosa* and (**b**) *Salvia pomifera*.

The methanolic extracts were prepared from the aerial parts of SF and SP. The polyphenols were detected in the extracts by HPLC coupled to electrospray quadrupole-time of flight mass spectrometry (HPLC–ESI-QTOF-MS) in negative ion mode. The base peak chromatograms of SF and SP extracts, displayed in Figure 2, show that the major phenolic components of SF were carnosol and carnosic acid, whereas SP was rich in 12-*O*-methylcarnosic acid.

The list of compounds identified in SF is given in Table 1. Identification of CA and rosmarinic acid was carried out by comparing their retention times and molecular masses with those of the commercial standards. Identification of other phenolics was based on a molecular formula derived from accurate mass determination, fragmentation pattern, and comparing the elution order on the reversed stationary phase with literature data. The HPLC-MS chromatogram of SF methanolic extract revealed the presence of three caffeic acid derivatives (caffeic acid, sagerinic acid, and rosmarinic acid), three flavonoid glycosides (luteolin-*O*-glucuronide, luteolin-*O*-glucoside, and apigenin-*O*-glucuronide), six flavonoid aglycones (nepetin, luteolin, apigenin, hispidulin, cirsimaritin, and genkwanin), seven abietane diterpenes (three rosmanol isomers, rosmadial, carnosol, CA, and 12-*O*-methylcarnosic acid), oleanolic/ursolic acid, and stearic acid.

### 2.2. The Presence of 12-O-Methylcarnosic Acid in SF and SP Is Proved by Its Fragmentation Pathway

**Figure 3:**
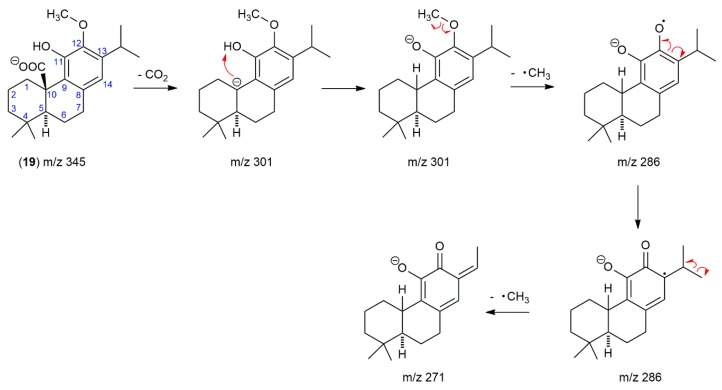
The suggested fragmentation of the deprotonated molecule of 12-*O*-methylcarnosic acid in the negative ion mode.

Compound 19, eluted in 17.5 min after CA (16.7 min), was observed in both SF and SP extracts. It produced a deprotonated molecule with *m*/*z* 345.2088, fitting molecular formula C_21_H_30_O_4_ and corresponding to methylated CA. The deprotonated molecule of *m*/*z* 345 gave a fragment with *m*/*z* 301 by loss of CO_2_, with further loss of methyl radical produced a base ion at *m*/*z* 286. The substance with molecular mass 346 and the same fragmentation pattern was previously identified as methyl carnosate (methyl ester of CA) in *S. officinalis* and SF [29,33,34,35] and *Rosmarinus officinalis* extracts [29,36]. Recently, 12-*O*-methylcarnosic acid, with the same retention behavior as “methyl carnosate”, was identified in the extract of SF using HPLC-SPE-NMR [28] and HPLC-MS [37]. The uncertainty about the identification of this peak by LC-MS is evident from the analysis of *Rosmarinus officinalis* and *S. officinalis* supercritical carbon dioxide extracts, where the peak with the similar retention, molecular mass, and fragmentation pattern was designed as “12-*O*-methylcarnosic acid, methyl carnosate” [38]. An ambiguous identification of compound 19 in literature and the fact, that the initial loss of carbon dioxide is substantially more probable from the deprotonated molecule (*m*/*z* 346) of 12-*O*-methylcarnosic acid than that of methyl carnosate led us to elucidate the compound 19 fragmentation pathway (Figure 3). The mapping of each step in fragmentation of compound 19 strongly supports the idea that the compound with molecular mass 346 and ion fragments *m*/*z* 301, 286, and 271 is 12-*O*-methylcarnosic acid. The fragmentation pathway starts with the elimination of carbon dioxide from deprotonated 12-*O*-methylcarnosic acid and is followed by proton transfer from phenolic hydroxyl to form a more stable anion (*m/z* 301); the negative charge is transferred from the carbon atom to the oxygen atom of the phenolate. Then, the methyl radical is released from the 12-methoxy group. The methyl radical elimination from methylated polyphenols is a common process because the phenolate anion (*m*/*z* 301) is converted to the resonance-stabilized anion-radical (m/z 286). Subsequently, the elimination of the second methyl radical from the isopropyl group may occur to form a new conjugated double bond and reinstate the closed-shell system (*m*/*z* 271 with low intensity) (Figure 3).

It should be noted that the initial step in fragmentation of deprotonated molecules of CA (*m*/*z* 331) and 12-*O*-methylcarnosic acid was the same, the loss of carbon dioxide. However, they differ in the next step of fragmentation, which can be explained by the different substituents at carbon C-12 and thus supports the structure of 12-*O*-methylcarnosic acid. An ion formed from CA by the loss of carbon dioxide (*m*/*z* 287) is fragmented to an ion radical (*m*/*z* 244) by the loss of the isopropyl radical from carbon C-13 (Table 1). This result can be easily explained because the homolytic cleavage of the side chain by removal of a relatively stable isopropyl radical is more probable than the release of a hydrogen atom from the hydroxyl group attached to carbon C-12. 

### 2.3. The Main Phenolic Substance of SP Is 12-O-Methylcarnosic Acid 

Figure 2 shows that the main phenolic component of the methanolic extract of SP aerial parts is 12-*O*-methylcarnosic acid. The list of phenolic compounds found in SP is given in Table 2. The HPLC-MS analysis of SP revealed 17 compounds including caffeic acid derivatives, flavonoid glycosides, flavonoid aglycones, abietane diterpenes, oleanolic/ursolic acid, and stearic acid.

The main difference in the composition of SP and SF is the presence of abietane diterpenes, which represent the main group of phenolic compounds in both SP and SF. The major phenolic components of SF were carnosol and CA. Surprisingly, carnosol and CA were not detected in SP as well as their degradation product rosmadial (Table 2). Unlike SF, SP contains danshensu (*syn*. 3,4-dihydroxyphenyllactic acid), hyperoside (quercetin 3-galactoside) and two not identified diterpenes (compounds 25 and 26).

### 2.4. SP and SF Extracts and CA Affect Cell Viability

**Figure 4:**
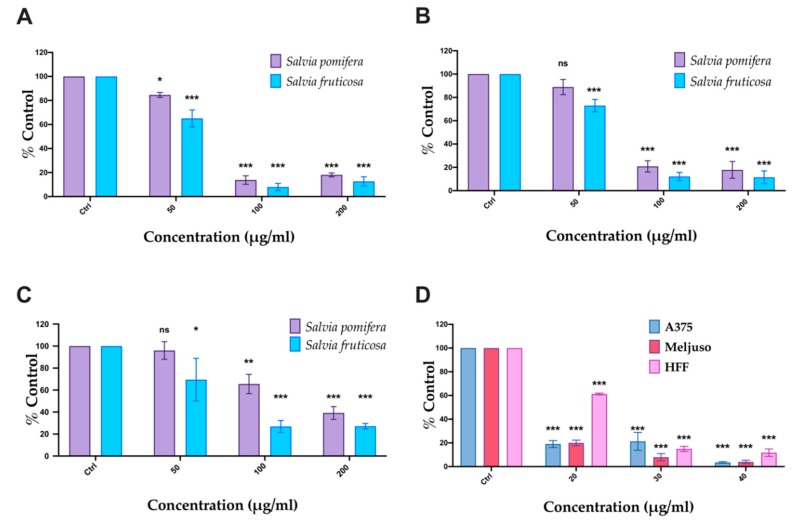
Antiproliferative activity of *Salvia fruticosa* Mill. (SF) and *Salvia pomifera* L. (SP) extracts evaluated by MTT assay. (**A**) A375, (**B**) Mel JuSo, and (**C**) human foreskin fibroblast (HFF) cells were incubated with SP or SF extracts and (**D**) carnosic acid (CA) for 48 h. The data are the means ± S.E.M. of three independent experiments; * < 0.05, ** < 0.01 and *** < 0.001.

The cell viability was studied using the MTT metabolic activity assay on two human melanoma cell lines (A375 and Mel JuSo) and human foreskin fibroblast (HFF) used as non-transformed control cells. Cells were incubated with SP and SF extracts (50, 100, 200 μg/mL) and CA (20, 30, 40 μg/mL) for 48 h. Both plant extracts and CA affected the metabolic activity in all cell lines tested in a dosage-dependent manner (Figure 4). As a negative control, DMSO corresponding to highest concentration in samples was used. SF extract had a more significant effect at lower concentrations (<70 μg/mL) compared to SP extract in both melanoma cell lines, A375 (Figure 4A) and Mel JuSo (Figure 4B) but also affects to some extent metabolic activity in HFF cells (Figure 4C). The IC_50_ values of SF and SP extracts were 57.95 μg/mL and 70.29 μg/mL for A375 cells and 63.57 μg/mL and 76.53 μg/mL for Mel JuSo cells. CA (Figure 4D) exhibited a significant antiproliferative effect to both melanoma cell lines at all concentrations tested, with an IC_50_ of 7.56 μg/mL and 11.75 μg/mL, respectively. Non-transformed HFF cells were less sensitive to CA, which was toxic at higher concentrations (>30 μg/mL) with an observed IC_50_ value equal to 21.87 μg/mL (Table 3).

**Figure 5:**
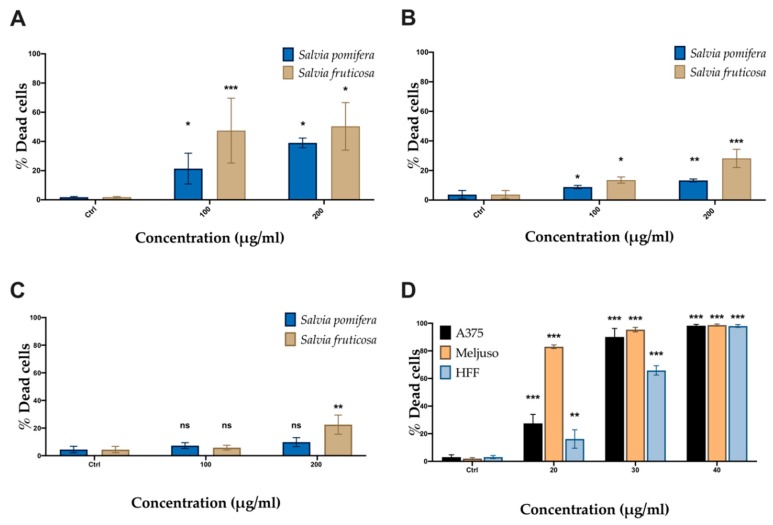
Antiproliferative activity of SF and SP extracts evaluated by PI-exclusion assay. (**A**) A375, (**B**) Mel JuSo, and (**C**) HFF cells were incubated with SP or SF extracts and (**D**) CA for 48 h. The data are the means ± S.E.M. of three independent experiments; * < 0.05, ** < 0.01 and *** < 0.001.

In addition to MTT assay, we also performed the PI-exclusion assay revealing death cells. A375 and Mel JuSo melanoma cells were incubated with SP and SF methanolic extracts and CA for 48 h. Based on data obtained from the MTT assay, we chose the concentrations of each extract or CA that showed significant inhibition of cell metabolic activity of melanoma cells, while in parallel exhibited lower toxicity in non-cancer HFF, i.e., 100 and 200 μg/mL. Using flow cytometry analysis we determined the percentage of PI-positive cells. The data show that A375 cells (Figure 5A) were more sensitive than Mel JuSo (Figure 5B). A slight increase in cell death was observed in Mel JuSo (Figure 5B) and an even slighter effect on non-cancer cells (HFF) (Figure 5C). Accordingly, to MTT results, the SF extract is able to induce cell death more efficiently compared to SP (Figure 5A–C). In all cases, the effect of these extracts on HFF cell viability was significantly lower (Figure 5C). CA was more efficient than the extracts (Figure 5D), indicating the possibility that CA is an active component of the SF extract. 

### 2.5. SP, SF Extracts and CA Arrest Cell Cycle

**Figure 6:**
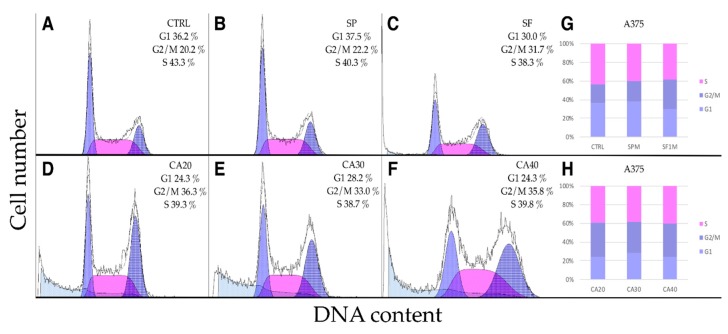
Cell cycle analysis of A375 cells. (**A**) Control DMSO treated cells. Cells treated 24 h with 100 μg/mL of (**B**) SP, (**C**) SF methanolic extracts or (**D**–**F**) CA at concentrations of 20, 30 and 40 μg/mL. (**G**,**H**) SF extract and to a larger extent CA significantly increased the proportion of A375 cells in G2/M phase.

Comparison of MTT and PI assays results showed that metabolic activity of cells was significantly reduced at concentrations 100 and 200 μg/mL but not all cells were dead, indicating cytostatic effect. Therefore, we studied whether the extracts and CA arrest the cell cycle. In A375 cells (Figure 6A–H), SP had no significant effect on the cell cycle, while SF slightly increased the proportion of G2/M phase (Figure 6B,C). This effect was more pronounced upon CA (20, 30 and 40 μg/mL) treatment, a lower proportion of G1 and higher of G2/M phase cells were observed. Upon CA treatment, also the sub-G1 phase representing death and apoptotic cells was apparent (Figure 6D–F). 

**Figure 7:**
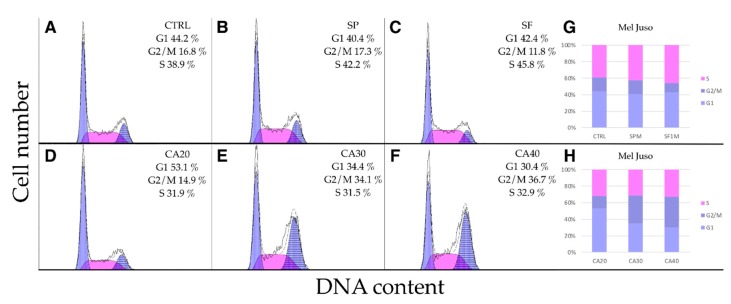
Cell cycle analysis of Mel JuSo cells. (**A**) Control DMSO treated cells. Cells treated 24 h with 100 μg/mL of (**B**) SP, (**C**) SF methanolic extracts or (**D**–**F**) CA at concentrations of 20, 30 and 40 μg/mL. (**G**,**H**) CA at concentrations 30 and 40 μg/mL significantly increased the proportion of Mel JuSo cells in G2/M phase.

**Figure 8:**
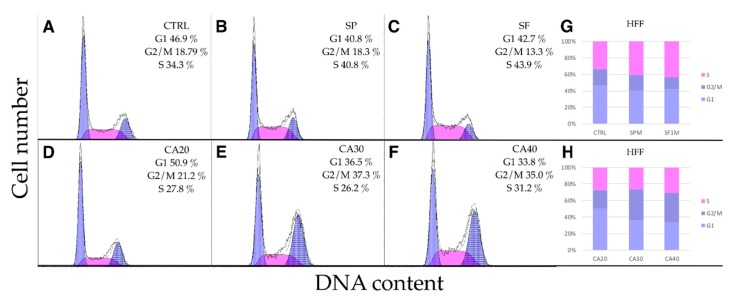
Cell cycle analysis of HFF cells. (**A**) Control DMSO treated cells. Cells treated 24 h with 100 μg/mL of (**B**) SP, (**C**) SF methanolic extracts or (**D**–**F**) CA at concentrations of 20, 30 and 40 μg/mL. (**G**,**H**) CA at concentrations 30 and 40 μg/mL significantly increased the proportion of HFF cells in G2/M phase.

Mel JuSo (Figure 7) and HFF (Figure 8) cells responded similarly but less extensively than A375. The extracts had no effect on cell cycle progression, while CA at concentrations of 30 and 40 μg/mL increased the proportion of cells in G2/M phase. Slight increase of S-phase cells was observed upon extract treatments. These results indicate that CA as a component of SF extract plays a crucial role in cell cycle arrest. 

### 2.6. CA Changes the Dynamics of Microtubules

**Figure 9:**
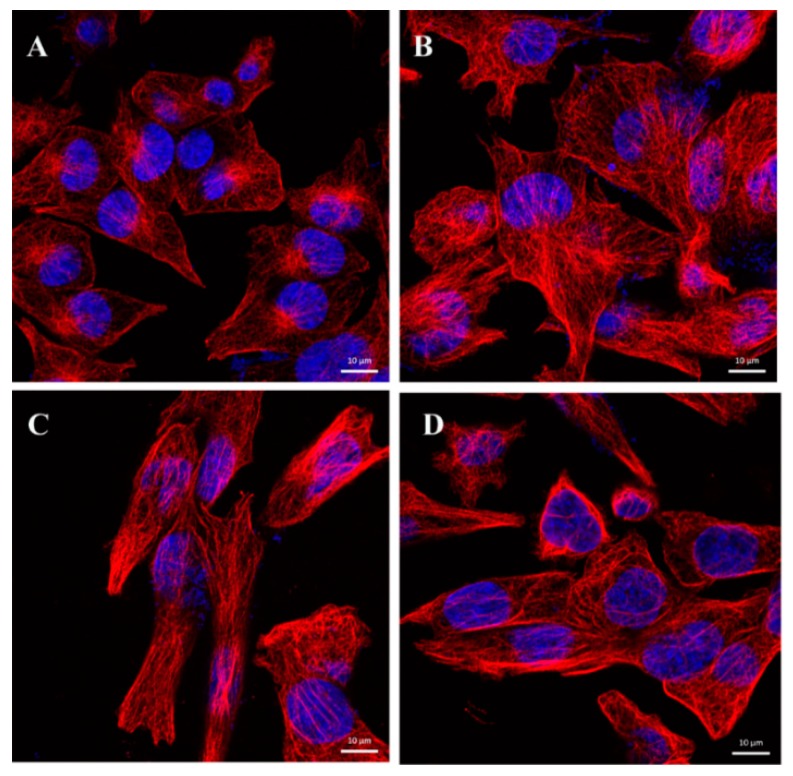
CA affects the dynamics of microtubules in A375 cells. Confocal laser scanning microscopy. Microtubules (red) stained by indirect immunofluorescence and nuclei (DAPI staining, blue) after 24 h treatment with CA at concentrations (**B**) 20, (**C**) 30 and (**D**) 40 μg/mL. The regular network of microtubules was observed in (**A**) control DMSO treated cells, while upon CA treatment thicker microtubule bundles and larger nuclei in enlarged cells were apparent (**B**–**D**). Bars = 10 μm.

The observed accumulation of cells in the G2/M phase of the cell cycle upon CA treatment is a typical feature of microtubule disruption. Therefore, we used indirect immunofluorescence to study the morphology of microtubules. Since the effect on cell cycle arrest was more pronounced in A375 cells treated with CA, we studied microtubule morphology after 24 h treatment of A375 cells with CA at concentrations 20, 30 and 40 μg/mL. Control cells showed a regular network of cytoplasmic microtubules (Figure 9A). Incubation of A375 cells with CA led to morphological changes of microtubules, which formed thick bundles resembling response to MT-stabilizing drugs such as taxanes (Figure 9B–D). The cells were enlarged with large nuclei. It is probable that this microtubule disorganization is the cause of the above-mentioned accumulation of cells at the G2/M phase.

## 3. Discussion 

In the present study, we investigated the biological effects of methanolic extracts of two *Salvia* species, *S. pomifera* L. and *S. fruticosa* Mill., on two melanoma cell lines (A375 and Mel JuSo) in comparison with non-cancer human fibroblast (HFF) cells. The composition of methanolic extracts of SF and SP aerial parts was investigated by HPLC–ESI–QTOF–MS. The negative ion mode was chosen because it is generally more suitable for the detection of plant polyphenols by ESI-MS due to the formation of a stable phenolate anion. A facile ionization of carboxylic group makes the negative mode even more advantageous for the detection of phenolic acids (e.g., caffeic acid, rosmarinic acid, and CA), which are the main antioxidants in *Salvia*. We found that the major diterpene phenolic compounds in SF were carnosol and CA; other major peaks on the chromatogram were 12-*O*-methylcarnosic acid and rosmarinic acid. Sagerinic acid and genkwanin were identified in SF for the first time. Many studies have reported differences in the composition of polyphenols in SF. Our detection of phenolic constituents seems to be in good agreement with the previous result obtained by HPLC-SPE-NMR, where flavones hispidulin, salvigenin, and cirsimaritin and the diterpenes CA, carnosol, and 12-*O*-methylcarnosic acid were identified as the major components of the SF extract [28]. A similar spectrum of diterpenes in SF was also reported by Topcu et al. [32]. The negative mode of ionization allowed the detection of nonpolar carboxylic acids with high retention times, oleanolic/ursolic acid and stearic acid. These nonpolar acids were previously found in SF [32], but not in SP.

To our knowledge, phenolic compounds in SP were investigated only by Cvetkovik et al. [39]. In their work, rosmarinic acid, salvianolic acid K, and a nonspecified caffeic acid derivative were reported by HPLC-ESI-MS using the negative ion mode. It means that we detected danshensu (*syn*. 3,4-dihydroxyphenyllactic acid), caffeic acid, hyperoside, luteolin-*O*-hexoside, sagerinic acid, rosmarinic acid, luteolin, apigenin, hispidulin, cirsimaritin, rosmanol, genkwanin, 12-*O*-methylcarnosic acid, and stearic acid in SP for the first time. The dominant peak in the HPLC-MS chromatogram was 12-*O*-methylcarnosic acid, whereas CA and carnosol, the main phenolic components of SF, were completely absent in SP. We reported here that the peak eluted after CA on the reversed stationary phase with molecular formula C_21_H_30_O_4_, producing typical fragments in the negative mode *m*/*z* 301 and 286, and widely detected in *Rosmarinus officinalis*, *S. officinalis*, and *S. fruticosa* as “methyl carnosate” [29,34,35,36] is 12-*O*-methylcarnosic acid, which is in good agreement with the previous finding of 12-*O*-methylcarnosic acid in *Rosmarinus officinalis* [37].

**Figure 10:**
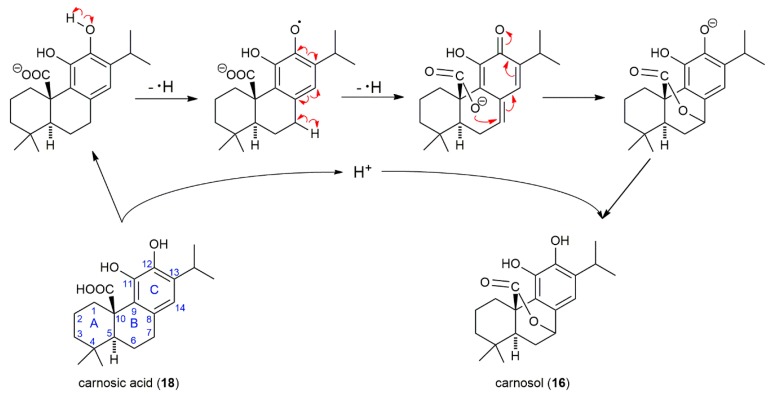
The suggested mechanism of CA oxidation to carnosol.

CA and carnosol, the two main phenolic diterpenes found in rosemary and sage, have received attention in food and medicinal chemistry because of their strong antioxidant activity. It was found previously that all plant species containing carnosol had also CA in their leaves [40] because CA (molecular mass 332) can be oxidized even non-enzymatically to carnosol (molecular mass 330) and next products as rosmanol and rosmadial [36]. We also tried to detect an oxidation product of 12-*O*-methylcarnosic acid analogous to carnosol formed from CA. The deprotonated molecule with *m*/*z* 343, the theoretical product of oxidation of 12-*O*-methylcarnosic acid, was not found by us in the extracted ion chromatogram of both SF and SP. Because 12-*O*-methylcarnosic acid is not susceptible to this type of oxidation, the 12-hydroxy group plays an important role in the oxidation of CA to carnosol. The exact mechanism for the oxidation of CA to carnosol is not known. It is supposed that the mechanism of CA oxidation to carnosol occurs in two steps. The first step is considered to be the oxidation to a quinone of CA followed by a non-specified isomerization to carnosol [41,42]. However, this predicted mechanism does not explain the activation of carbon C-7 for carboxyl group attack, the mode of isomerization of quinone to carnosol, and regeneration of the ortho-diphenol group. We suggested the detailed mechanism, which is initiated by the formation of a semiquinone by subtracting of the hydrogen atom from the phenolic group attached to carbon C-12, followed by oxidative removal of a hydrogen atom from carbon C-7 resulting in the formation of a quinonoid structure stabilized by resonance. The subsequent nucleophilic attack of the carboxylate to carbon C-7 is driven by restoring an aromatic character of the ring C (Figure 10).

The SF extract affected cell viability and proliferation more significantly than the SP extract. In order to evaluate whether CA is the compound that is responsible for SF extract higher toxicity, we also studied the effect of CA itself. We found that CA affects cancer cells even more efficiently than SF extracts. These results suggest that CA might be one of the most important compounds responsible for the more significant effect of SF extracts. Thus, our presumption is in accordance with the results of several other authors studying the effect of CA on cancer cells. CA is a natural polyphenolic diterpene widely used in traditional medicine, which has several effects on cancer cells. It was described that CA affects proliferation of tumor cells of various origins such as leukemia, liver, colorectal and brain cancer [16,17,18,19,20], showed the antiproliferative effect of CA on human and rat lung fibroblasts and lung cancer cells, while rosmarinic acid which is also component of *Salvia* extracts had no effect, but potentiated the effect of CA [17]. Also, Xavier et al. [9] did not observe the significant antiproliferative activity of rosmarinic acid on colorectal carcinoma cells and assumed that other more effective components are in the extracts [9]. It is clear that in whole extracts all compounds and their interactions are involved in biological activity. There are several pieces of evidence that CA is a molecule of interest for further studies concerning use in cancer therapy. In addition, the effects of CA alone or in combination with anti-cancer drugs on melanoma cells were described. Lin et al., 2018 [43] demonstrated the antiproliferative effect of CA in combination with carmustine or lomustine in B16F10 melanoma cells and tumors arising from B16F10 cells xenografted to the back of C57BL/6 mice. Since CA induces NQO1 expression in melanoma cells in vitro, it affects the cell proliferation and sensitizes melanoma cells to other antitumor drugs [12]. Furthermore, it has been shown that CA inhibits the adhesion and migration of melanoma cells at least partially due to the inhibition of epithelial-mesenchymal transition (EMT) and AKT inactivation [16,18]. 

Our results are in alignment with the above-discussed data as we demonstrated that CA affects cell proliferation and the cell death of two melanoma cell lines. In addition, we showed that SF extract and CA affect the distribution of cell cycle phases; more specifically, we observed an increased proportion of the G2/M phase. Since microtubules are involved in anaphase chromosome segregation, we checked the morphology of them using indirect immunofluorescence. Comparing to control, we observed thicker microtubule bundles in cells treated with CA. This could be a consequence of the disruption of microtubule dynamics by CA, which prevents the entry of cells into mitosis. Stabilization of microtubules is the general mode of action of taxanes, the important group of anti-cancer drugs [44]. G2/M arrest of cell cycle accompanied by a reduction in cyclin A levels was previously described in colorectal cancer Caco-2 cells upon CA treatment [45]. Contrary other authors demonstrated cell cycle arrest at G0/G1 upon CA treatment on B16F10 melanoma cells [43]. In spite of the fact that the SP extract did not contain CA, it also exhibited antiproliferative activity nevertheless, indicating that additional *Salvia* components have antiproliferative activity. It is likely that rosmarinic acid, a phenolic compound found in several *Salvia* species [39], or other phenolic and terpenic compounds found in SP extracts exert antiproliferative activity in a similar manner to CA. It is obvious that antiproliferative activities were properties of the whole extracts, very likely resulting from multi-factorial effects, which involve most of their components. Nevertheless, SP extracts exhibited a significantly lower effect on cell viability compared to CA alone or the SF extract containing CA and had almost no effect on cell cycle arrest indicating a key role of CA in cell cycle influencing. Another interesting point of our study is that Mel JuSo cells were less sensitive to plant extracts or CA compared to A375 cells. These differences in the sensitivity could be explained by differences in the genetic background of the cell lines tested. A375 is a melanoma cell line, which harbors an activating BRAF^V600E^ mutation while a characteristic NRAS activating mutation exists in Mel JuSo cells. In accordance with our observation, other authors suggested different cellular response dependent on mutations of the MAPK/ERK pathway genes. The colon cancer cell lines were more or less resistant to *Salvia* water extracts based on their genetic profile [9]. In more details, KRAS mutated cells were more sensitive to *Salvia* extracts, while the same compounds did not have any antiproliferative effect in BRAF mutated cells. On the contrary, in our study, we observed that NRAS cells are more resistant to *Salvia* extracts than BRAF^V600E^ cells. These discrepancies could reflect the capacity of melanoma cells to activate the BRAF protein in an NRAS-independent manner as well as that these melanoma cell lines are more dependent on the MAPK/ERK activation pathway compared to colorectal cells, where the predominant pathway seems to be the PI3K/AKT pathway. Furthermore, it has been already described that SF water extracts induce apoptosis in breast cancer cell lines by inhibiting the MAPK/ERK pathway [2]. Alternatively, several studies have shown that the activity of CA and *Salvia* extracts is cell line and cancer type-dependent [8,13,46,47,48]; for example, only HER2^+^ breast cancer cell lines growth is affected while HER2^-^ cells are completely resistant to CA.

Previous studies have pointed out the difficulty of comparing results obtained from many different methods of extraction, and that a more consistent approach should be performed in order to properly validate the effect of *Salvia* extracts in cancer cells [3]. For example, *S. lanigera* water extracts exhibited lower toxicity [7] compared to acetone extracts from the same plant. In addition, the results and mechanism of action may vary significantly among different *Salvia* species and cell lines used. These differences could be minimized by performing experiments with precise doses of pure biologically active constituents extracted from plants. On the other hand, the use of whole extracts could shed light upon possible synergisms between *Salvia* components. Further studies would explore which are the most active components of *Salvia* extracts and whether constituents of extracts are interesting for melanoma treatment as monotherapy or in combination with other anticancer drugs.

## 4. Materials and Methods 

### 4.1. Plant Material and Extract Preparation

Dried aerial parts of *Salvia pomifera* L. (SP) and *Salvia fruticosa* Mill. (SF) were kindly provided by the natural cosmetics company Apivita, Greece. Plants were cultivated and collected in Crete, Greece. The aerial parts of both plants were harvested from 3-years-old plants at the optimal ontogenetic phase just before flowering in 2014, cultivated by a local producer on a medium-scale plot of sand-clay soil. Plant material was examined and identified by the herbarium at the Department of Pharmacognosy and Botany, Faculty of Pharmacy, Bratislava, Slovakia. Voucher specimens have been deposited as well (SP-2014, SF1-2014). SP and SF plants were air-dried and pulverized in laboratory and particle size were adjusted using a No. 355 sieve. The extracts were obtained after treating powdered leaves (5 g) with 100 mL methanol using a Soxhlet extractor for 6 h and subsequently filtered with filter paper no 1. Finally, the extract was evaporated until complete dryness using a rotary evaporator and vacuum suction at 40 °C under reduced pressure conditions. The concentrated crude extract was weighed and stored at 4 °C. 

### 4.2. Identification of Compounds by Liquid Chromatography-Mass Spectrometry

(LC-Q/TOF-MS/MS) A Dionex Ultimate 3000RS HPLC system (ThermoScientific, California, CA, USA) connected to a Bruker MicrOTOF-Q II mass spectrometer (Bruker Daltonics, Bremen, Germany) was used. Extracts (100 µg/mL; 10 µl) were separated on a 2.1 mm × 150 mm, 5 μm Dionex Acclaim 120 C8 column (ThermoScientific, California, CA, USA), protected by a guard column with a flow rate of 0.3 mL/min at 23 °C. The mobile phase was composed of water containing 0.1% (*v*/*v*) acetic acid (Sigma Aldrich, Prague, Czech Republic) (A) and acetonitrile (Sigma Aldrich, Prague, Czech Republic) (B). Ultra-pure grade water was prepared by a Milli-Q system (Millipore, Massachusetts, MA, USA). The following elution program was employed: a linear gradient from 10 to 80% of eluent B in 15 min, it was kept at these conditions for 5 min, and followed by equilibration for another 2 min. The effluent was monitored at 254 nm and directly introduced into the Q/TOF mass spectrometer equipped with an ESI source. Instrument control and data acquisition were achieved by the Compass 1.3 (HyStar 3.2) software. MS experiments were performed in the negative electrospray ionization mode, the capillary voltage was maintained at 4350 V with the endplate offset at −500 V. Nitrogen was used as the drying and nebulizing gas at a flow rate of 6 L/min and a pressure of 2.0 bar, respectively. Mass spectra were recorded in a range from 50 to 2000 *m*/*z*, at a rate of 1.0 Hz. The mass spectrometer was calibrated using 10 mM sodium formate in 50% (*v*/*v*) isopropyl alcohol at the beginning of each LC run with a 20 μL loop flush. This step ensured the mass accuracy was below 5 ppm across the entire chromatogram. High-resolution MS and MS/MS spectra were first investigated to obtain the elemental formula of each compound. The final identification of target compounds relied on isotope pattern matching with a combination of MS/MS and retention behavior.

### 4.3. Cell Lines and Culture Conditions

The NRAS mutated (Mel JuSo) and the BRAF^V600E^ mutated (A375) human malignant melanoma cell lines were obtained from ECACC, Salisbury, UK. A third cell line derived from human foreskin fibroblasts (HFF) was obtained from ATCC. Both melanoma cell lines were cultivated in RPMI-1640 while HFF cell line in DMEM medium (HyClone Laboratories, Inc., Utah, UT, USA). In all experiments performed, 2 mM l-glutamine (PAA Laboratories, Austria), 10% (*v*/*v*) fetal calf serum, 100 IU/mL penicillin and 100 μg/mL streptomycin (HyClone Laboratories) were additionally added to the culture medium. Cells were cultivated at 37 °C under 5% (*v*/*v*) CO_2_ in a high-humidity-atmosphere and were split every 48 h.

### 4.4. MTT Cell Proliferation Assay

Cells were seeded in 96-well plates (Nunc A/S, Roskilde, Denmark) at a concentration of 10^4^ cells/mL in 200 μL of the medium in conditions as described above (37 °C, 5% CO_2_). After 24 h of incubation, cells were treated with *Salvia* extracts (at concentrations 50, 100 and 200 μg/mL) and CA (at concentrations 20, 30 and 40 μg/mL) for additional 48 h and finally MTT assays were performed as previously described by Hammerova et al. [49]. Optical density was measured at all times at 570 nm using an SLT Spectra-Shell-Microplate Reader (SLT-Lab Instruments GmBH, Saltsburg, Austria). Each concentration of a compound was examined in four replicate wells and each experiment was repeated three times.

### 4.5. Propidium Iodide (PI) Exclusion Assay

Cells were seeded in 24-well tissue culture test plates (Orange Scientific, Braine-l’Alleud, Brabant wallon, Belgium) at a concentration of 5 × 10^4^ cells/mL in 300 μL of the medium in conditions as described above (37 °C, 5% CO_2_). After 24 h *Salvia* extracts or CA were added for an additional 48 h. Finally, cells were harvested, washed in PBS twice and PI staining was performed as previously described [49]. The percentage of dead (PI-positive) cells was measured using the Cytomics FC 500 flow cytometer (Beckman Coulter) using FL3 channel (emission wavelength 620 nm). 10,000 cells per sample were acquired, each sample was analyzed in triplicate and each experiment was repeated three times.

### 4.6. Cell Cycle Analysis

Cells were seeded at a specific concentration of 0.5 × 10^6^ cells/mL in 6-well plates and cultivated till 90% confluence was reached. Then, cells were incubated with *Salvia* extracts or CA for additional 24 h. Finally, cells were treated with trypsin and harvested into ice-cold PBS, fixed in 70% (*v*/*v*) ethanol for 30 min on ice and processed as previously described [50]. In order to evaluate cell cycle phases, 10,000 cells per sample were initially acquired by Cytomics FC 500 flow cytometer using FL3 channel (emission wavelength 620 nm) and data were further analyzed by using software Multicycle AV for Windows (Phoenix Flow system, San Diego, California, CA, USA). Each sample was analyzed in triplicates and each experiment was repeated three times.

### 4.7. Indirect Immunofluorescence and DAPI Staining

A375 cells, grown 24 h on coverslips in the presence of CA (20, 30 and 40 μg/mL), were fixed with 3% (*w*/*v*) paraformaldehyde in PBS for 20 min at room temperature, permeabilized with 0.2% (*w*/*v*) Triton X-100 in PBS for 5 min and preincubated for 20 min in 0.5% (*w*/*v*) BSA in PBS. Cells were stained with primary anti-β-tubulin (TU-01, Exbio, Prague, Czech Republic) antibody overnight. Alexa Fluor 594 Donkey Anti-Mouse IgG (A 21203, Life Technologies) was used as the secondary antibody. DAPI at a final concentration of 1 μg/mL was added to the solution of the secondary antibody for visualization of nuclei. Slides were observed using a confocal microscope (LSM 700, Carl Zeiss GmbH, Jena, Germany).

### 4.8. Statistics

Statistical analysis was carried out using the statistical program GraphPad Prism 8.0.2(159), San Diego, CA 92108, USA. Results were analyzed with student t-test and the threshold for statistical significance was set to *p* < 0.01 and *p* < 0.05.

## 5. Conclusions

In this study, 13 phenolic compounds were detected in the methanolic extract of *S. pomifera* for the first time using HPLC-ESI-MS/MS analysis and the composition of the methanolic extracts of *S. fruticosa* and *S. pomifera* were compared. CA and its metabolite carnosol were the most abundant phenolic compounds in SF, while they were completely absent in SP. The main phenolic constituent of SP was 12-*O*-methylcarnosic acid and its mass/mass fragmentation pathway in the negative ion mode was explained. The detailed mechanism of CA oxidation to carnosol, based on the resistance of 12-*O*-methylcarnosic acid to this type of oxidation, was also suggested. In addition, the antiproliferative activity of these extracts and CA on two melanoma cell lines (A375 and Mel JuSo) in comparison with non-cancer human fibroblast (HFF) cells were demonstrated. The first evidence that CA affects microtubule dynamics and arrests cell cycle of melanoma cells in the G2/M phase was provided. The effects of SF extract and CA were more significant than the effect of SP extract indicating that CA could be the primary compound responsible for the cytostatic effect of SF extracts. 

## Figures and Tables

**Table 1:** Compounds Identified in the Methanolic Extract of *Salvia fruticosa*.

Compound No.	RT min	Measured Mass of [M – H]¯	Calculated Mass of [M – H]¯	Error in ppm	Major Fragment ions	Formula	Tentative Identification	Ref.
**1**	5.90	179.0346	179.0344	1.12	135	C_9_H_8_O_4_	Caffeic acid	[22]
**2**	6.80	461.0722	461.0720	0.43	285	C_21_H_18_O_12_	Luteolin-*O*-glucuronide	[23]
**3**	6.90	447.0921	447.0927	1.34	285	C_21_H_20_O_11_	Luteolin-*O*-glucoside	[22,24]
**4**	7.60	445.0774	445.0771	0.67	269; 113	C_21_H_18_O_11_	Apigenin-*O*-glucuronide	[23]
**5**	7.70	719.1537	719.1612	10.4	161; 323; 279; 197; 179; 135	C_36_H_32_O_16_	Sagerinic acid ^a^	[25]
**6**	7.70	359.0774	359.0767	1.95	161; 197; 179; 135	C_18_H_16_O_8_	Rosmarinic acid	[22,24,26]
**7**	9.30	315.0509	315.0505	1.27	300	C_16_H_12_O_7_	Nepetin	[27]
**8**	9.40	285.0433	285.0399	11.9	No fragment	C_15_H_10_O_6_	Luteolin	[22,24,26]
**9**	10.4	269.0473	269.0450	8.55	225; 201; 159; 151; 149; 117; 107	C_15_H_10_O_5_	Apigenin	[22,24,26]
**10**	10.4	299.0562	299.0556	2.01	284; 228; 137	C_16_H_12_O_6_	Hispidulin	[28]
**11**	11.7	313.0730	313.0712	5.75	283; 298; 255	C_17_H_14_O_6_	Cirsimaritin	[28]
**12**	12.0	345.1731	345.1702	8.40	301; 283	C_20_H_26_O_5_	Rosmanol isomer	[29]
**13**	12.4	345.1724	345.1702	6.37	283	C_20_H_26_O_5_	Rosmanol isomer	[29]
**14**	12.8	345.1737	345.1702	10.1	283; 301	C_20_H_26_O_5_	Rosmanol isomer	[29]
**15**	12.8	283.0623	283.0607	5.65	268; 240	C_16_H_12_O_5_	Genkwanin ^a^	[30,31]
**16**	15.2	329.1771	329.1753	5.47	285	C_20_H_26_O_4_	Carnosol	[28,32]
**17**	15.7	343.1583	343.1546	10.8	315; 299; 287; 244	C_20_H_24_O_5_	Rosmadial	[32]
**18**	16.7	331.1933	331.1909	7.25	287; 244	C_20_H_28_O_4_	Carnosic acid	[28,32]
**19**	17.5	345.2088	345.2066	6.37	286; 301; 271	C_21_H_30_O_4_	12-*O*-Methylcarnosic acid	[28,32]
**20**	19.1	455.3530	455.3525	1.10	No fragment	C_30_H_48_O_3_	Oleanolic and ursolic acid	[32]
**21**	20.8	283.2661	283.2637	8.47	No fragment	C_18_H_36_O_2_	Stearic acid	[32]

^a^ identified in *S. fruticosa* for the first time.

**Table 2:** Compounds Detected in the Methanolic Extract of *Salvia pomifera*.

Compound no.	RT min	Measured Mass of [M – H]¯	Calculated Mass of [M – H]¯	Error in ppm	Major Fragment Ions	Formula	Tentative Identification	Ref.
**23**	2.80	197.0451	197.0450	0.51	135; 123; 179; 151; 109	C_9_H_10_O_5_	Danshensu ^a^	[33]
**1**	5.90	179.0354	179.0344	5.59	135	C_9_H_8_O_4_	Caffeic acid ^a^	[22]
**24**	6.20	463.0879	463.0877	0.43	301; 135	C_21_H_20_O_12_	Hyperoside ^a^	[34]
**3**	6.90	447.0944	447.0927	3.80	285	C_21_H_20_O_11_	Luteolin-*O*-hexoside ^a^	[22]
**5**	7.70	719.1634	719.1612	3.06	161; 323	C_36_H_32_O_16_	Sagerinic acid ^a^	[25]
**6**	7.70	359.0773	359.0767	1.67	161; 197; 135	C_18_H_16_O_8_	Rosmarinic acid	[22,24,26]
**8**	9.40	285.0411	285.0399	4.21	133	C_15_H_10_O_6_	Luteolin ^a^	[22,24,26]
**9**	10.4	269.0465	269.0450	5.58	151; 135; 117	C_15_H_10_O_5_	Apigenin ^a^	[22,24,26]
**10**	10.4	299.0559	299.0556	1.00	284; 269; 212; 165; 136	C_16_H_12_O_6_	Hispidulin ^a^	[28]
**11**	11.7	313.0717	313.0712	1.60	283; 298; 269; 255	C_17_H_14_O_6_	Cirsimaritin ^a^	[28]
**13**	12.4	345.1706	345.1702	1.16	283; 299; 268	C_20_H_26_O_5_	Rosmanol isomer ^a^	[29]
**15**	12.8	283.0607	283.0607	0.00	268	C_16_H_12_O_5_	Genkwanin ^a^	[30,31]
**25**	13.7	317.2122	317.2117	1.58	233; 299	C_20_H_30_O_3_	Not identified diterpene	
**26**	14.4	345.2069	345.2066	0.87	299; 316; 283; 263; 249	C_21_H_30_O_4_	Not identified diterpene	
**19**	17.5	345.2066	345.2066	0.00	286; 301; 271	C_21_H_30_O_4_	12-*O*-Methylcarnosic acid ^a^	[28,32]
**20**	19.1	455.3527	455.3525	0.44	No fragment	C_30_H_48_O_3_	Oleanolic and ursolic acid	[32]
**21**	20.8	283.2639	283.2637	0.71	No fragment	C_18_H_36_O_2_	Stearic acid ^a^	[32]

^a^ identified in *S. pomifera* for the first time.

**Table 3:** The Cytotoxicity of *Salvia* Methanolic Extracts and Carnosic Acid (CA). Results Present IC_50_ Values After 48 h of Treatment, A375, Mel Juso, and Human Foreskin Fibroblast (HFF) Cells.

Extract (μg/mL) Compound (μg/mL)	A375	Mel JuSo	HFF
***Salvia pomifera* (SF)**	70.29	76.53	153.5
***Salvia fruticosa* (SP)**	57.95	63.57	72.67
**Carnosic acid (CA)**	7.56	11.75	21.87

## References

[1] Boukhary R., Raafat K., Ghoneim A.I., Aboul-Ela M., El-Lakany A. (2016). Anti-inflammatory and antioxidant activities of *Salvia fruticosa*: An HPLC determination of phenolic contents. Evid.-Based Complement. Altern. Med..

[2] Tundis R., Iacopetta D., Sinicropi M.S., Bonesi M., Leporini M., Passalacqua N.G., Ceramella J., Menichini F., Loizzo M.R. (2017). Assessment of antioxidant, antitumor and pro-apoptotic effects of *Salvia fruticosa* Mill. subsp. thomasii (Lacaita) Brullo, Guglielmo, Pavone & Terrasi (Lamiaceae). Food Chem. Toxicol..

[3] Duletić-Laušević S., Alimpić Aradski A., Šavikin K., Knežević A., Milutinović M., Stević T., Vukojević J., Marković S., Marin P.D. (2018). Composition and biological activities of Libyan *Salvia fruticosa* Mill. and *S. lanigera* Poir. extracts. S. Afr. J. Bot..

[4] Gali-Muhtasib H. (2006). Anticancer and medicinal properties of essential oil and extracts of East Mediterranean sage (*Salvia triloba*). Adv. Phytomed..

[5] Ramos A.A., Azqueta A., Pereira-Wilson C., Collins A.R. (2010). Polyphenolic compounds from *Salvia* species protect cellular DNA from oxidation and stimulate DNA repair in cultured human cells. J. Agric. Food Chem..

[6] Fraihat A., Alatrash L., Abbasi R., Abu-Irmaileh B., Hamed S., Mohammad M., Abu-Rish E., Bustanji Y. (2018). Inhibitory effects of methanol extracts of selected plants on proliferation of two human melanoma cell lines. Trop. J. Pharm. Res..

[7] Alimpic A.Z., Kotur N., Stanković B., Marin P.D., Matevski V., Al Sheef N., Duletić-Laušević S. (2015). The in vitro antioxidative and cytotoxic effects of selected *Salvia* species water extracts. J. Appl. Bot. Food Qual..

[8] Abu-Dahab R., Afifi F., Kasabri V., Majdalawi L., Naffa R. (2012). Comparison of the antiproliferative activity of crude ethanol extracts of nine *Salvia* species grown in Jordan against breast cancer cell line models. Pharmacogn. Mag..

[9] Xavier C.P.R., Lima C.F., Fernandes-Ferreira M., Pereira-Wilson C. (2009). *Salvia fruticosa*, *Salvia officinalis*, and rosmarinic acid induce apoptosis and inhibit proliferation of human colorectal cell lines: The role in MAPK/ERK pathway. Nutr. Cancer.

[10] Ramos A.A., Pedro D., Collins A.R., Pereira-Wilson C. (2012). Protection by *Salvia* extracts against oxidative and alkylation damage to DNA in human HCT15 and CO115 cells. J. Toxicol. Env. Heal Part A Curr. Issues.

[11] Stagos D., Portesis N., Spanou C., Mossialos D., Aligiannis N., Chaita E., Panagoulis C., Reri E., Skaltsounis L., Tsatsakis A.M. (2012). Correlation of total polyphenolic content with antioxidant and antibacterial activity of 24 extracts from Greek domestic Lamiaceae species. Food Chem. Toxicol..

[12] Arakawa N., Okubo A., Yasuhira S., Takahashi K., Amano H., Akasaka T., Masuda T., Shibazaki M., Maesawa C. (2018). Carnosic acid, an inducer of Nad(P)H quinone oxidoreductase 1, enhances the cytotoxicity of β-lapachone in melanoma cell lines. Oncol. Lett..

[13] Petiwala S.M., Johnson J.J. (2015). Diterpenes from rosemary (*Rosmarinus officinalis*): Defining their potential for anti-cancer activity. Cancer Lett..

[14] Birtić S., Dussort P., Pierre F.X., Bily A.C., Roller M. (2015). Carnosic acid. Phytochemistry.

[15] Barni M.V., Carlini M.J., Cafferata E.G., Puricelli L., Moreno S. (2012). Carnosic acid inhibits the proliferation and migration capacity of human colorectal cancer cells. Oncol. Rep..

[16] Bahri S., Jameleddine S., Shlyonsky V. (2016). Relevance of carnosic acid to the treatment of several health disorders: Molecular targets and mechanisms. Biomed. Pharmacother..

[17] Bahri S., Mies F., Ben Ali R., Mlika M., Jameleddine S., Mc Entee K., Shlyonsky V. (2017). Rosmarinic acid potentiates carnosic acid induced apoptosis in lung fibroblasts. PLoS ONE.

[18] Park S.Y., Song H., Sung M.K., Kang Y.H., Lee K.W., Park J.H.Y. (2014). Carnosic acid inhibits the epithelial-mesenchymal transition in B16F10 melanoma cells: A possible mechanism for the inhibition of cell migration. Int. J. Mol. Sci..

[19] Pesakhov S., Nachliely M., Barvish Z., Aqaqe N., Schwartzman B., Voronov E., Sharoni Y., Studzinski G.P., Fishman D., Danilenko M. (2016). Cancer-selective cytotoxic Ca^2+^ overload in acute myeloid leukemia cells and attenuation of disease progression in mice by synergistically acting polyphenols curcumin and carnosic acid. Oncotarget.

[20] Kar S., Palit S., Ball W.B., Das P.K. (2012). Carnosic acid modulates Akt/IKK/NF-κB signaling by PP2A and induces intrinsic and extrinsic pathway mediated apoptosis in human prostate carcinoma PC-3 cells. Apoptosis.

[21] Domingues B., Lopes J., Soares P., Populo H. (2018). Melanoma treatment in review. ImmunoTargets Ther..

[22] Sarrou E., Martens S., Chatzopoulou P. (2016). Metabolite profiling and antioxidative activity of Sage (*Salvia fruticosa* Mill.) under the influence of genotype and harvesting period. Ind. Crops Prod..

[23] Moharram F.A., Mahmoud I.I., Mahmoud M.R., Sabry S. (2006). Polyphenolic profile and biological study of *Salvia fruticosa*. NPC Nat. Prod. Commun..

[24] Vergine M., Nicolì F., Negro C., Luvisi A., Nutricati E., Annunziata Accogli R., Sabella E., Miceli A. (2019). Phytochemical profiles and antioxidant activity of *Salvia* species from southern Italy. Rec. Nat. Prod..

[25] Lu Y., Foo L.Y. (1999). Rosmarinic acid derivatives from *Salvia officinalis*. Phytochemistry.

[26] Exarchou V., Nenadis N., Tsimidou M., Gerothanassis I.P., Troganis A., Boskou D. (2002). Antioxidant activities and phenolic composition of extracts from Greek oregano, Greek sage, and summer savory. J. Agric. Food Chem..

[27] Lee S.H., Kim H.W., Lee M.K., Kim Y.J., Asamenew G., Cha Y.S., Kim J.B. (2018). Phenolic profiling and quantitative determination of common sage (*Salvia plebeia* R. Br.) by UPLC-DAD-QTOF/MS. Eur. Food Res. Technol..

[28] Exarchou V., Kanetis L., Charalambous Z., Apers S., Pieters L., Gekas V., Goulas V. (2015). HPLC-SPE-NMR characterization of major metabolites in *Salvia fruticosa* Mill. extract with antifungal potential: Relevance of carnosic acid, carnosol, and hispidulin. J. Agric. Food Chem..

[29] Kontogianni V.G., Tomic G., Nikolic I., Nerantzaki A.A., Sayyad N., Stosic-Grujicic S., Stojanovic I., Gerothanassis I.P., Tzakos A.G. (2013). Phytochemical profile of *Rosmarinus officinalis* and *Salvia officinalis* extracts and correlation to their antioxidant and anti-proliferative activity. Food Chem..

[30] Ayatollahi S.A., Shojaii A., Kobarfard F., Mohammadzadeh M., Choudhary M.I. (2009). Two flavones from *Salvia leriaefolia*. Iran. J. Pharm. Res..

[31] Cuvelier M.E., Richard H., Berset C. (1996). Antioxidative activity and phenolic composition of pilot-plant and commercial extracts of sage and rosemary. JAOCS J. Am. Oil Chem. Soc..

[32] Topçu G., Öztürk M., Kuşman T., Demirkoz A.A.B., Kolak U., Ulubelen A. (2013). Terpenoids, essential oil composition, fatty acid profile, and biological activities of Anatolian *Salvia fruticosa* Mill. Turk. J. Chem..

[33] Liu A.H., Guo H., Ye M., Lin Y.H., Sun J.H., Xu M., Guo D.A. (2007). Detection, characterization and identification of phenolic acids in danshen using high-performance liquid chromatography with diode array detection and electrospray ionization mass spectrometry. J. Chromatogr. A.

[34] Šulniūtė V., Pukalskas A., Venskutonis P.R. (2017). Phytochemical composition of fractions isolated from ten *Salvia* species by supercritical carbon dioxide and pressurized liquid extraction methods. Food Chem..

[35] Pizzale L., Bortolomeazzi R., Vichi S., Überegger E., Conte L.S. (2002). Antioxidant activity of sage (*Salvia officinalis* and *S fruticosa*) and oregano (*Origanum onites* and *O. indercedens*) extracts related to their phenolic compound content. J. Sci. Food Agric..

[36] Rangarajan R., Dodbiba E., Smuts J.P., Lang J.C., Zhang Y., Armstrong D.W. (2012). Degradation study of carnosic acid, carnosol, rosmarinic acid, and rosemary extract (*Rosmarinus officinalis* L.) assessed using HPLC. J. Agric. Food Chem..

[37] Munné-Bosch S., Alegre L., Schwarz K. (2000). The formation of phenolic diterpenes in *Rosmarinus officinalis* L. under Mediterranean climate. Eur. Food Res. Technol..

[38] Ivanović J., Dilas S., Jadranin M., Vajs V., Babović N., Petrović S., Žižović I. (2009). Supercritical carbon dioxide extraction of antioxidants from rosemary (*Rosmarinus officinalis* L.) and sage (*Salvia officinalis* L.). J. Serb. Chem. Soc..

[39] Cvetkovikj I., Stefkov G., Acevska J., Stanoeva J.P., Karapandzova M., Stefova M., Dimitrovska A., Kulevanova S. (2013). Polyphenolic characterization and chromatographic methods for fast assessment of culinary *Salvia* species from South East Europe. J. Chromatogr. A.

[40] Abreu M.E., Müller M., Alegre L., Munné-Bosch S. (2008). Phenolic diterpene and α-tocopherol contents in leaf extracts of 60 *Salvia* species. J. Sci. Food Agric..

[41] Masuda T., Inaba Y., Takeda Y. (2001). Antioxidant mechanism of carnosic acid: Structural identification of two oxidation products. J. Agric. Food Chem..

[42] Loussouarn M., Krieger-Liszkay A., Svilar L., Bily A., Birtić S., Havaux M. (2017). Carnosic acid and carnosol, two major antioxidants of rosemary, act through different mechanisms. Plant Physiol..

[43] Lin K.-I., Lin C.-C., Kuo S.-M., Lai J.-C., Wang Y.-Q., You H.-L., Hsu M.-L., Chen C.-H., Shiu L.-Y. (2018). Carnosic acid impedes cell growth and enhances anticancer effects of carmustine and lomustine in melanoma. Biosci. Rep..

[44] Cristina C. (2014). Rohena and Susan, L. Mooberry Recent progress with microtubule stabilizers: New compounds, binding modes and cellular activities. Nat. Prod. Rep..

[45] Visanji J.M., Thompson D.G., Padfield P.J. (2006). Induction of G2/M phase cell cycle arrest by carnosol and carnosic acid is associated with alteration of cyclin A and cyclin B1 levels. Cancer Lett..

[46] Xiang Q., Ma Y., Dong J., Shen R. (2015). Carnosic acid induces apoptosis associated with mitochondrial dysfunction and Akt inactivation in HepG2 cells. Int. J. Food Sci. Nutr..

[47] Gao Q., Liu H., Yao Y., Geng L., Zhang X., Jiang L., Shi B., Yang F. (2015). Carnosic acid induces autophagic cell death through inhibition of the Akt/mTOR pathway in human hepatoma cells. J. Appl. Toxicol..

[48] Akaberi M., Mehri S., Iranshahi M. (2015). Multiple pro-apoptotic targets of abietane diterpenoids from *Salvia* species. Fitoterapia.

[49] Hammerová J., Uldrijan S., Táborská E., Slaninová I. (2011). Benzo[c]phenanthridine alkaloids exhibit strong anti-proliferative activity in malignant melanoma cells regardless of their p53 status. J. Dermatol. Sci..

[50] Slanina J., Pachnikova G., Čarnecka M., Porubova Koubikova L., Adamkova L., Humpa O., Šmejkal K., Slaninova I. (2014). Identification of key structural characteristics of *Schisandra chinensis* lignans involved in P-glycoprotein inhibition. J. Nat. Prod..

